# Draft Genome Sequences of Three Strains of a Novel *Rhizobiales* Species Isolated from Forest Soil

**DOI:** 10.1128/genomeA.01452-17

**Published:** 2018-02-01

**Authors:** Grace Pold, Marcel Huntemann, Manoj Pillay, Natalia Mikhailova, Dimitrios Stamatis, T. B. K. Reddy, Chris Daum, Nicole Shapiro, Nikos Kyrpides, Tanja Woyke, Kristen M. DeAngelis

**Affiliations:** a Graduate Program in Organismic and Evolutionary Biology, University of Massachusetts–Amherst, Amherst, Massachusetts, USA; b U.S. Department of Energy Joint Genome Institute, Walnut Creek, California, USA; c Department of Microbiology, University of Massachusetts–Amherst, Amherst, Massachusetts, USA

## Abstract

Three strains of a novel *Rhizobiales* species were isolated from temperate deciduous forest soil in central Massachusetts. Their genomes consist of 9.09 to 10.29 Mb over 3 to 4 scaffolds each and indicate that diverse nitrogenous compounds are used by these organisms.

## GENOME ANNOUNCEMENT

Although the alphaproteobacterial order *Rhizobiales* is often associated with nitrogen fixation, substantial phenotypic and genotypic variations are increasingly being uncovered (1). We isolated three *Rhizobiales* sp. strains from a long-term soil-warming experiment (2) in order to continue the characterization of this dominant and diverse order.

*Rhizobiales* sp. strains GAS113, GAS188, and GAS191 were isolated under aerobic conditions using VL55 medium (3) with 0.005% xylan (GAS191), mixed plant polymers (GAS113), and yeast extract and peptone (GAS188). Colonies appeared after 16 to 23 days of growth and were selected for genomic sequencing based on their slow growth (0.01 h^−1^) and prolonged lag times in liquid media. These Gram-negative bacteria grow between a pH of 5.5 and 7.5 and up to a temperature of 30°C.

DNA was extracted from 6-week-old colonies on R2A plates using the Qiagen Genomic-tip protocol. Whole-genome sequencing was completed at the Joint Genome Institute (JGI) on the PacBio RS platform (4). The reads were assembled using the Hierarchical Genome Assembly Process (HGAP) version 2.3.0 (5), resulting in genomes of 9.09 to 10.29 Mb over 3 to 4 scaffolds.

Gene prediction and functional annotation were performed using the DOE-JGI annotation pipeline (6, 7). Of the 8,370 to 9,736 coding sequences, 75.5 to 77.2% were assigned a function and 8.3% were predicted to contain signal peptides using SignalP (8). The genomes contain 51 to 54 tRNAs and a single rRNA operon. The genomes are available for comparative analysis through the Integrated Microbial Genomes (IMG) data management system (9).

Based on the sequence of their 16S rRNA gene and whole-genome sequences, these novel isolates appear to form a sister clade to methylotrophic *Methylocystis* species (10), sharing >97% average nucleotide identity (ANI) and >99% 16S identity with each other but <73% ANI and <94% 16S identity with the closest members in the family *Methylcystaceae* (10, 11). Genomes of the three new isolates lack the methane monooxygenase found in *Methylocystis* species and instead show an expansion of genes for amino acid transport and metabolism (Clusters of Orthologous Groups [COG] category E; 15.8% of reads assigned to COGs versus 6.7% in *Methylocystis* genomes), secondary metabolite biosynthesis, transport and catabolism (COG category Q; 3.9% versus 2.2%), and carbohydrate transport and metabolism (COG category G; 6.9% versus 3.3%). dbCAN (12) predicts this suite of carbohydrate-active enzymes to contain 20 classes of glycoside hydrolases and be particularly rich in GH109 (glycoprotein and glycolipid degradation). Combined with a large number of amino acid and peptide transporters annotated in the genome and a paucity of monosaccharide uptake genes, the genome indicates that these bacteria derive a substantial fraction of their resources from low concentrations of nitrogenous compounds characteristic of soil organic matter. The presence of a complete coenzyme F420 biosynthesis pathway may also indicate an enhanced competitive ability under low redox or highly biostatic conditions (11).

### Accession number(s).

These whole-genome shotgun projects have been deposited at GenBank under the accession numbers FNLG00000000 (GAS113), FNSS00000000 (GAS188), and FNSX00000000 (GAS191). The versions described in this paper are the first versions, FNLG01000000, FNSS01000000, and FNSX01000000, respectively.
